# Supplementary material for: Distribution and Restoration of Serotonin-Immunoreactive Paraneuronal Cells During Caudal Fin Regeneration in Zebrafish
Source: Front Mol Neurosci. 2019 Sep 19;12:227. doi: 10.3389/fnmol.2019.00227 (PMC6763699; doi:10.3389/fnmol.2019.00227)
Supplement: Supplementary file 1 [file Image_1.pdf]

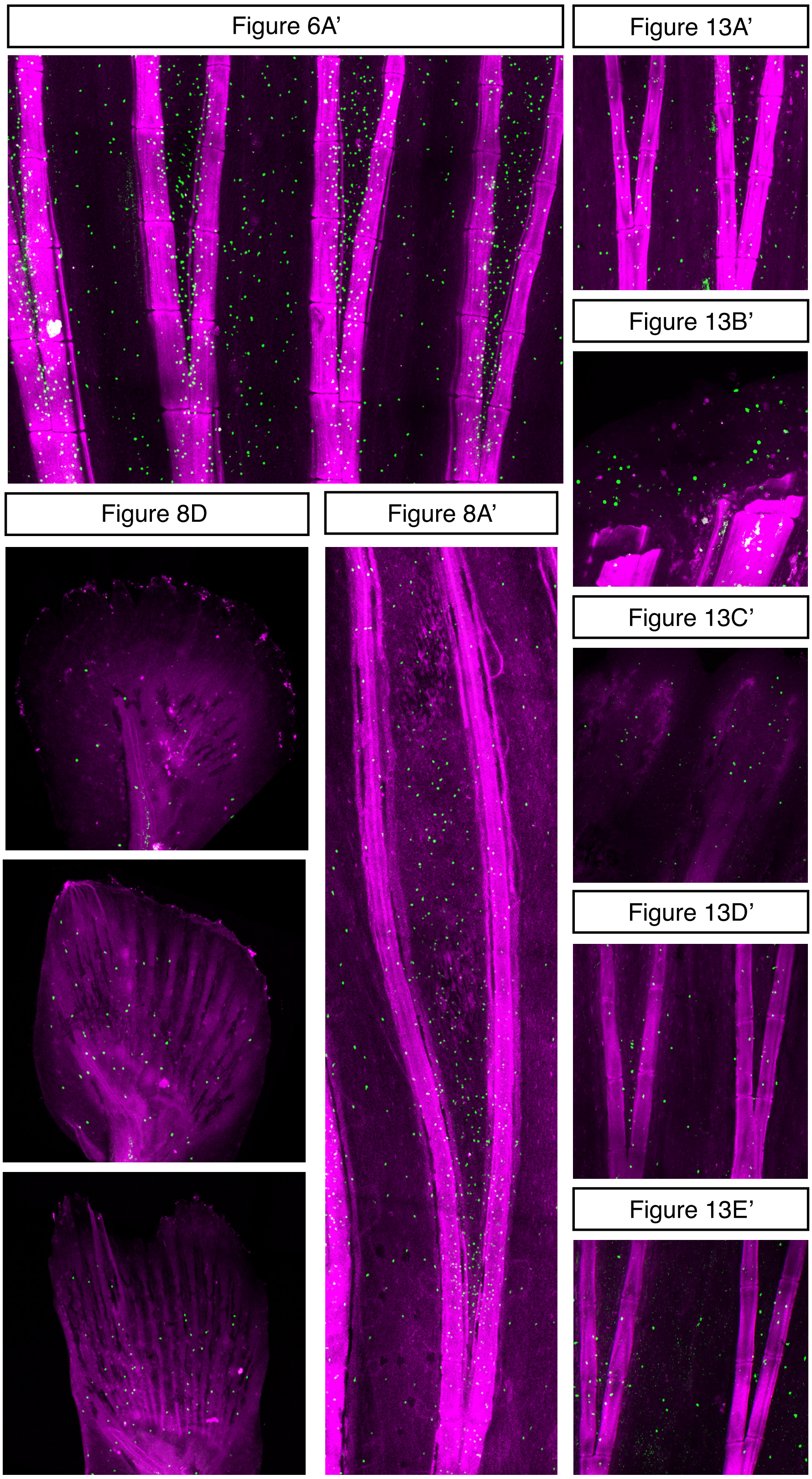

Supplementary Figure S1. Images of Zooms from main figures without white cell-projection dots.
The corresponding main figures are indicated above the image. Tissue Autofluorescence in pink. Serotonin-positive cells in green.
